# The Association Between Radiographic and MRI Cervical Spine Parameters in Patients With Down Syndrome

**DOI:** 10.7759/cureus.25046

**Published:** 2022-05-16

**Authors:** Masayoshi Machida, Brett Rocos, Katsuaki Taira, Naho Nemoto, Noboru Oikawa, Tomonori Kinoshita, Takashi Kozu, Kazuyoshi Nakanishi

**Affiliations:** 1 Department of Orthopaedic Surgery, Saitama Children Medical Center, Saitama, JPN; 2 Department of Orthopaedic Surgery, Bart's Health NHS Trust, London, GBR; 3 Department of Orthopaedic Surgery, Nihon University, Tokyo, JPN

**Keywords:** spine instability, c-spine, spine imaging, pediatric spine, spine alignment

## Abstract

Introduction

Many patients with Down syndrome (DS) develop upper cervical spine instability that may lead to spinal cord injury. The purpose of this study was to investigate the association between the spinal cord compression in MRI and the occipto-cervical instability evident on plain radiographs in a Japanese population.

Methods

A retrospective analysis of cervical spine radiographs and MRI acquired from patients with DS was performed. Radiographic evaluation included measuring the atlanto-dental interval (ADI) and space available for the cord. The basion axial interval (BAI) and Weisel-Rothman (WR) measurements were taken to quantify occipto-axial (OA) and atlanto-occipital (AO) instability. These parameters were collected in patients both with (positive) and without (negative) spinal cord compression evident on MR imaging in a neutral position and the values were compared. In addition, we investigated the association between spinal cord compression and previously defined abnormal values with logistic regression analysis (abnormal values: ADI>6mm, SAC<14mm, BAI<-12mm or >5mm in neutral position).

Results

There were 17 patients in the positive group and 52 patients in the negative group. WR was 7.4 mm±6.0 in positive group and 8.6 mm±4.8 in negative group (p=0.31) in neutral position, 3.9 mm±5.4 and 6.3±5.0 (p=0.06) in flexion, and 7.0 mm±6.8 and 7.2 mm±4.8 (p=0.75) in extension, respectively. The difference in WR between flexion and extension was 3.1 mm ± 4.6 and 0.9 mm ± 3.8, respectively (p=0.15). All other parameters showed significant differences between the two groups excluding BAI in extension (p<0.05). In addition, abnormal values that significantly correlated with cord compression were ADI (odds ratio 42.3 p<0.01 95% CI 4.16-430.0) and SAC (odds ratio 31.90 p=0.013 95% CI 2.06-494.0).

Conclusions

These data suggest that OA and AA instability measured with ADI, SAC, and BAI are significantly associated with spinal cord compression in MRI; whereas instability measured with WR and DWR is not. In addition, the previously defined abnormal thresholds for the ADI and SAC can be used for screening the Japanese population.

## Introduction

Down syndrome (DS) is a common genetic variation with a range of phenotypes, including some of which affect the spine. Up to 60% of affected patients develop instability of the atlanto-axial (AA) or occipito-cervical (OC) spine because of underlying collagen defects [1-6]. Furthermore, 1% of patients will show signs and symptoms of neurological deficit, including quadriplegia [7-9]. Unfortunately, the cognitive dysfunction that affects these patients makes neurological assessment unreliable, so imaging studies to evaluate the presence and severity of cervical instability are essential at an early stage.

There are several metrics used to radiologically assess AA and OC instability. These include the atlanto-dental interval (ADI), space available for the cord (SAC) at C1, and basion axial interval (BAI). Unfortunately, none of these measures were designed specifically to either a paediatric or a DS population [4]. In these cases, a more valid measurement used to assess proximal cervical instability is the Weisel-Rothman (WR) measurement, which compares basion-C1 horizontal distance measured on flexion and extension lateral cervical radiographs [10]. Bouchard et al. defined thresholds for radiological screening for instability in lateral plain radiographs (ADI>6mm, SAC<14mm, BAI<-12mm or >5mm in neutral position). Patients meeting these criteria should be considered being at risk for cervical spine instability and be further investigated using cross-sectional imaging [4].

While the above values are useful, the associations between these values and the incidence of cord compression in patients with DS are unknown. Understanding these associations are essential in managing the risk of conservative management, counselling patients and families and managing the neck during anaesthesia. The aim of this study was to characterise the association between the spinal cord compression in magnetic resonance imaging (MRI) and the OC instability evident on plain radiographs and to evaluate whether above abnormal values of OC instability apply in a Japanese population.

This article was previously presented as a poster at the 2021 Japan Pediatric Orthopaedic Association Annual Meeting on December 2, 2021.

## Materials and methods

Local research ethical board approval was granted for the study. A retrospective analysis of the radiographic investigations acquired for all patients diagnosed with DS in a single centre between 2010 and 2021 was carried out. Patients were included if diagnosed with DS and had both lateral cervical radiographs and MR imaging spanning occiput to C3. Patients were excluded if DS was not confirmed, patients presented with trauma or incomplete imaging was unavailable.

**Figure 1:**
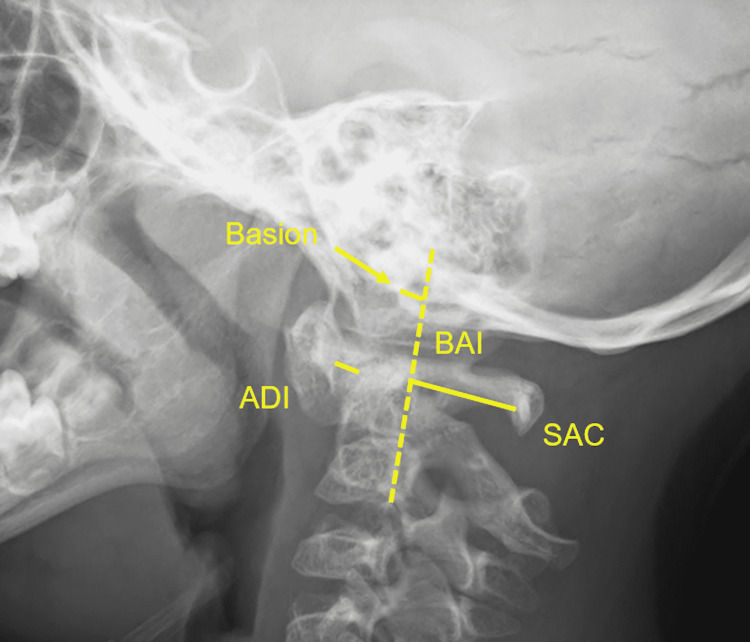
Demonstration of atlanto-dental interval (ADI), space available for cord at C1 (SAC), and basion axial interval (BAI) measurements. Note the basion is anterior to the line tangent to the posterior body of C2.

**Figure 2:**
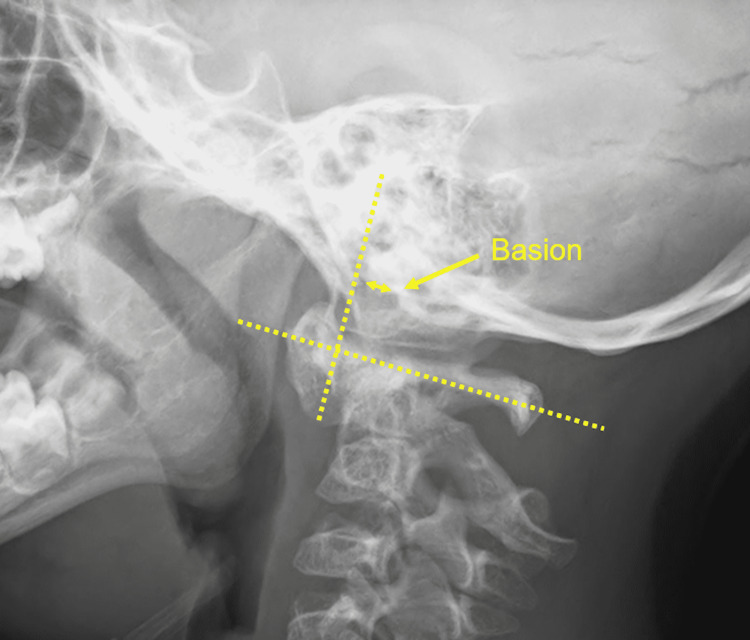
Demonstration of the difference in Wiesel-Rothman (DWR), the difference in measurement between flexion and extension views of the distance between the basion and a line tangent to the posterior edge of the anterior arch of C1.

Each radiograph was reviewed by both a spine surgeon and radiologist. The ADI, SAC at C1, BAI and WR in both flexion and extension were recorded (Figures 1, 2). The difference in WR between flexion and extension (DWR) was then calculated. BAI was used to quantify occipto-axial (OA) instability and WR to evaluate atlanto-occipital (AO) instability. A negative BAI value was recorded when the basion was positioned anterior to the posterior axial line and a positive value if the basion was posterior to this line. A negative WR value was recorded when the basion was positioned anterior to the line perpendicular to the C1 axial, tangent to the posterior edge of the anterior ring.

The MR examinations were performed with the neck in a neutral position using an MR scanner (PHILIPS®, Intera, 1,5 T, The Netherlands). The presence of cord compression was determined by both a spine surgeon and radiologist, and recorded as a binary value using both radiological signs of vertebral instability and the presence of cord signal change. The BAI and WR for each group either with (positive) and without (negative) spinal cord compression evident on MR imaging in neutral position were then compared.

The data were analyzed using R (version 3.6.1, The R Foundation for Statistical Computing, Vienna, Austria). Variables are presented as means, standard deviation (SD), and ranges. A Shapiro-Wilk test was conducted to assess distribution of continuous variables, and a Mann-Whitney U test was used for variables with non-normal distributions. Logistic regression analyses for patient characteristics were perform to adjust for concurrent effects of various factors. For all statistical tests, a p value <0.05 was considered significant.

## Results

The group showing cord compression with or without cord signal change (positive group) included 17 patients (five males) (Table 1). No patients were excluded for incomplete imaging. The mean age was 8.0±1.7 years (2.5-18.5 years). The group not showing cord compression (negative group) comprised 52 patients (19 males). The mean age was 6.7±4.8 (1.6-16.5 years).

**Table 1:** The patient demographics

	Positive group (N=17)	Negative group (N=52)
Age (years)	8.0±1.7 (range: 2.5-18.5 years)	6.7±4.8 (range: 1.6-16.5 years)
Male	5	19
Female	12	33

ADI was 7.9 mm±2.7 in the positive group and 3.6 mm±1.7 in the negative group (p<0.01) in a neutral position, 8.3 mm±2.5 and 4.3 mm±1.9 in flexion (p<0.01), and 5.0 mm± 3.3 and 2.5 mm±1.1 in extension (p<0.01), respectively (Table 2). SAC was 11.1 mm±3.1 in the positive group and 15.4 mm±4.3 in the negative group (p<0.01) in a neutral position, 10.5 mm±3.7 and 15.2 mm±4.2 in flexion (p<0.01), and 14.1 mm± 4.1 and 16.5 mm±3.6 in extension (p<0.01), respectively. BAI was -9.4 mm±9.1 in the positive group and -2.4 mm±3.8 in the negative group (p<0.01) in a neutral position, -10.6 mm±10.2 and -6.2 mm±4.1 in flexion (p<0.01), respectively. WR was 3.9 mm±5.4 in the positive group and 6.3 mm±5.0 in the negative group (p=0.06) in flexion.

**Table 2:** Comparison of the radiographic parameters between the two groups ADI: atlanto-dental interval; SAC: space available for the cord at C1; BAI: basion axial interval; WR: Weisel-Rothman measurement; DWR: difference Weisel-Rothman measurement; *Statistically significant at p<0.05

		Positive group (N=17)	Negative group (N=52)
ADI (mm)	Neutral*	7.9±2.7	3.6±1.7
	Flexion*	8.3±2.5	4.3±1.9
	Extension*	5.0±3.3	2.5±1.1
SAC (mm)	Neutral*	11.1±3.1	15.4±4.3
	Flexion*	10.5±3.7	15.2±4.2
	Extension*	14.1±4.1	16.5±3.6
BAI (mm)	Neutral*	-9.4±9.1	-2.4±3.8
	Flexion*	-10.6±10.2	-6.2±4.1
	Extension	-5.9±10.1	-2.2±5.0
WR (mm)	Neutral	7.4±6.0	8.6±4.8
	Flexion	3.9±5.4	6.3±5.0
	Extension	7.0± 6.8	7.2±4.8
DWR (mm)		3.1±4.6	0.9±3.8

DWR was 3.1 mm±4.6 in positive group and 0.9 mm±3.8 in negative group (p=0.15). Table 3 shows the adjusted risks of cord compression with abnormal values for each radiographic parameter (abnormal values: ADI>6mm, SAC<14mm, BAI<-12mm or >5mm in neutral position). The factors that significantly correlated with cord compression on MRI were ADI (odds ratio 42.3 p<0.01 95% CI 4.16-430.0) and SAC (odds ratio 31.90 p=0.013 95% CI 2.06-494.0) (Table 3).

**Table 3:** The adjusted risks of cord compression with abnormal values for each radiographic parameter ADI: atlanto-dental interval, SAC: space available for the cord at C1; BAI: basion axial interval; *Statistically significant (p<0.05).

	Odds ratio	p-value	95% CI
ADI>6mm	42.30	<0.01*	4.16-430.00
SAC<14mm	31.90	0.013*	2.06-494.00
BAI < -12mm or > 5mm	31.9	0.13	0.37-2220.00

## Discussion

These data suggest that OA and AA instability measured with ADI, SAC and BAI are significantly associated with spinal cord compression in MRI; whereas instability measured with WR and DWR is not.

In previous reports, ADI >6 mm and SAC <14 mm in the neutral position were found to be associated with abnormal MRI findings [11-12]. Bouchard et al. defined abnormal values of ADI, SAC and BAI in the neutral position as a risk factor for abnormal MRI in a Canadian population [4]. Our results confirm that the definitions of abnormal ADI and SAC apply to Japanese patients with a diagnosis of DS for screening in logistic regression.

Hypermobility of the AO junction is observed in up to 60% of individuals with DS and is usually not associated with neurological risk [3]. There are many studies that describe abnormal O-C1 movement, defined by abnormal WR; however, none of these patients showed clinically relevant myelopathy on close clinical and radiographic evaluation [4]. In contrast, DS subjects with AO instability showed a significant reduction in the anterior subarachnoidal space in the flexed position on MRI, which is an important correlate to spinal cord damage [13]. To further clarify the understanding of this phenomenon, it may necessary to evaluate AO instability associated with spinal cord compression with MRI in flexion.

Currently, both the Special Olympics and the American Academy of Pediatrics recommend plain radiography as a screening method for instability [14]. However, Cremers et al. concluded that activity limitations and radiographic screening in asymptomatic children with DS might not be necessary [15]. We recommend the use of x-ray as a screening tool for assessing OA and AA bony instability, however, assessing AO instability with WR appears to be insufficient to indicate occipital-cervical fixation.

There are limitations in this study in addition to its retrospective design. These include the difficulty in evaluating the cranio-cervical junction represents using plain radiographs because of multiple superimposed structures, and the associated problems of interrater reliability and radiographic technique.

## Conclusions

These data show that ADI and SAC measured on lateral cervical radiographs are positively correlated to cord compression on MR imaging in patients with DS, whereas measurement of AO instability using the WR is not. Although the correlation with BAI did not meet statistical significance, it may be that this value would correlate in a larger sample. As a result, dynamic compression should be evaluated using flexion-extension MR imaging. In addition, the previously reported abnormal ADI and SAC can be reasonably applied for screening in the Japanese population.
